# Antenatal depressive symptoms and maternal health care utilisation: a population-based study of pregnant women in Ethiopia

**DOI:** 10.1186/s12884-016-1099-1

**Published:** 2016-10-10

**Authors:** Tesera Bitew, Charlotte Hanlon, Eskinder Kebede, Girmay Medhin, Abebaw Fekadu

**Affiliations:** 1 Department of Psychiatry, College of Health Sciences School of Medicine, Addis Ababa University, Addis Ababa, Ethiopia; 2 Department of Psychology, College of Social Science and Humanities, Debre Markos University, Debre Markos, Ethiopia; 3 Global Mental Health, Centre for Global Mental Health, Health Services and Population Research Department, Institute of Psychiatry, King’s College London, London, UK; 4 Department of Obstetrics and Gynecology, College of Health Sciences, Addis Ababa University, Addis Ababa, Ethiopia; 5 Aklilu Lemma Institute of Pathobiology, Addis Ababa University, Addis Ababa, Ethiopia; 6 Center for Innovative Drug Development and Therapeutic Trials for Africa (CDT-Africa), Addis Ababa University, Addis Ababa, Ethiopia; 7 Department of Psychological Medicine, Centre for Affective Disorders, Institute of Psychiatry, Psychology and Neuroscience, King’s College London, London, UK

**Keywords:** Maternal health care use, Antenatal care utilization, Antenatal care, Antenatal depression, Maternal depression, Sub-Saharan Africa, Ethiopia

## Abstract

**Background:**

Depressive symptoms during pregnancy can have multiple adverse effects on perinatal outcomes, including maternal morbidity and mortality. The potential impact of antenatal depressive symptoms on maternal health care use, however, has been little explored in low and middle-income countries (LMICs). This paper investigates whether maternal health care utilisation varies as a function of antenatal depressive symptoms.

**Methods:**

In a population-based cross-sectional survey, 1311 women in the second or third trimesters of pregnancy were recruited in Sodo district, Gurage Zone, southern Ethiopia. Depressive symptoms were measured using a locally validated version of the Patient Health Questionnaire (PHQ-9). The association between antenatal depressive symptoms and number of antenatal care (ANC) visits was examined using Poisson regression and the association of depression symptoms with emergency health care visits using negative binomial regression. Binary logistic regression was used to investigate the association of depressive symptoms with initiation, frequency and adequacy of antenatal care.

**Results:**

At PHQ-9 cut off of five or more, 29.5 % of participants had depressive symptoms. The majority (60.5 %) of women had attended for one or more ANC visits. Women with depressive symptoms had an increased risk of having more non-scheduled ANC visits (adjusted Risk Ratio (aRR) = 1.41, 95 % CI: 1.20, 1.65), as well as an increased number of emergency health care visits to both traditional providers (aRR = 1.64, 95 % CI: 1.17, 2.31) and biomedical providers (aRR = 1.31, 95 % CI: 1.04, 1.69) for pregnancy-related emergencies. However, antenatal depressive symptoms were not significantly associated with initiation of ANC.

**Conclusions:**

Increased non-scheduled ANC and emergency health care visits may be indicators of undetected depression in antenatal women, and have the potential to overwhelm the capacity and resources of health care systems, particularly in LMICs. Establishment of a system for detection, referral and treatment of antenatal depression, integrated within existing antenatal care, may reduce antenatal morbidity and treatment costs and promote efficiency of the health care system.

## Background

Maternal mortality remains a major public health challenge despite the encouraging changes achieved through the Millennium Development Goals [1, 2]. Nearly 99 % of global maternal deaths occur in low- and middle-income countries [1]. High maternal mortality is attributed to low antenatal health service utilization [3–7], high rates of home delivery [8–12] and low use of family planning services [1, 13]. Ethiopia contributes to between 3 and 5 % of global maternal mortality [5, 14]. Within Ethiopia, approximately three-quarters of these deaths are considered to be the result of undetected and untreated antenatal causes of obstetric complications, such as hemorrhage [15], infection [15], unsafe abortion, hypertension [15] and risk factors for obstructed labor [16–18]. To reduce the risk of pregnancy and obstetric complications, the World Health Organization recommends that women should attend at least four antenatal visits during pregnancy and deliver at a health facility [1].

In the African region, between 2000 and 2009, only 47 % of births were attended by skilled personnel and only 44 % of pregnant women had at least four antenatal care visits [19]. The situation is of major concern in Ethiopia [3, 4, 8, 11, 20–22], where only 11.7 % of births are attended by skilled health personnel, only 34 % of pregnant women attend for the recommended number of antenatal care visits [5] and there has been inadequate reduction in maternal mortality [23]. The total number of disability adjusted life years (DALYs) attributed to maternal mortality (10.6 %) in a rural area of Ethiopia exceeded that of malaria (10.4 %), tuberculosis (7 %), depression (6.5 %) or HIV (3.5 %) [24].

Maternal mortality and morbidity are established global public health challenges, but evidence is also emerging of the public health importance of maternal depression [25–30]. Antenatal depression is estimated to affect about 20 % of pregnant women in LMICs [31–35], compared to about 10 % in high-income countries [36, 37]. The adverse effects of maternal depression on productivity of mothers, child growth, health and behavioral outcomes, as well as perinatal outcomes, have been well documented [38–40].

Antenatal depressive symptoms have the potential to impact negatively upon health service utilization and thereby contribute to increased perinatal complications and maternal mortality. However, studies investigating the impact of antenatal depressive symptoms on health service utilization have been small in number and conducted almost exclusively in high-income countries. In these studies, antenatal depression has been found to be associated with increased unscheduled gynecological and obstetrician visits [25, 41], although no association was seen with initiation of antenatal care visits [42]. In studies examining the adequacy of antenatal care utilization, defined as attendance for 50 % or more of the expected ANC reviews [41, 43], no association was found between antenatal depression and adequate use of ANC. In the only study from Africa that the authors are aware of, a non-significant association was found between antenatal depression and antenatal care attendance [44].

There is, therefore, a clear gap in understanding the impact of depressive symptoms on antenatal care use, particularly in LMICs where perinatal outcomes are poor. Improved detection and treatment of antenatal depression is not prioritized in most LMIC health systems [45] but may improve ANC use, and therefore obstetric outcomes. Indeed, better evidence is needed to support greater priority to mental health care. In this paper, we report findings from a study that aimed to investigate whether antenatal depressive symptoms are associated with initiation, frequency and adequacy of ANC visits, and adherence with recommended schedules of ANC visits, in a rural Ethiopian setting. We hypothesised that ANC utilisation would vary as a function of antenatal depressive symptoms.

## Methods

The study was a cross-sectional, population-based survey.

### Study setting

The study was conducted in the Sodo District, Gurage Zone, Southern Nations, Nationalities and People’s Region (SNNPR) of Ethiopia, located about 100 km from the capital, Addis Ababa. The district is administratively divided into 58 kebeles or sub-districts (54 rural and four urban), the smallest administrative unit in Ethiopia. Sodo has a varied topography (40 % plains, 7 % mountainous, 30 % undulating and 23 % valleys) and agriculture is the main mode of subsistence in the area (Tafesse, 2002, personal communication). The population of the district was estimated to be 161,952 persons (79,356 men; 82,596 women) in 2007 [46]. The majority of the inhabitants belong to the Sodo Gurage ethnic group (85 %) [47, 48]. The remaining population are mostly Oromo and Amhara in ethnicity [48]. The official language of the region and the district is Amharic.

### Sample size estimation and recruitment of participants

A total of 1311 women were recruited into the study between September and November 2014. Eligibility criteria for women were as follows: (1) in the second or third trimester of pregnancy; (2) continuous residence in the area for at least the preceding 6 months; (3) not having hearing or cognitive impairment to the extent of impairing capacity to communicate adequately, and (4) giving informed consent to take part in the study.

EpiInfo version 7 software (EpiInfo; CDC, 2000) was used to estimate the sample size assuming a statistical power of 80 % with a two tailed 5 % margin of error for three dependent variables (ANC use, delivery care use and pregnancy complications). ANC utilization as the dependent variable yielded the greatest sample size: 276 women with depressive symptoms, the exposure or independent variable, assuming 34 % antenatal care utilization [5] among the unexposed and a 6 % difference between exposed and unexposed groups. To obtain 276 pregnant women with antenatal depressive symptoms, assuming a prevalence of 19.9 % [32], 1387 pregnant women needed to be screened for depression.

### Locating participants

In the Ethiopian health system, community-based health workers (Health Extension Workers; HEWs) are responsible for health prevention and promotion activities. HEWs collect community-based data for the health information system, especially information about maternal health. During their 3-monthly house-to-house visits of the population living in their sub-district, they are expected to identify and monitor pregnant mothers and to keep accurate and up-to-date maternal records in the health posts (primary care facilities at the sub-district level). In addition, the health development army (a community-based network of health education volunteers, each of whom covers five families) are required to report pregnant women in their respective units to the HEWs. For this study, HEWs, health development army leaders, community leaders and pregnant women themselves acted as key informants to identify all pregnant women living in their sub-district. The data collectors then carried out home visits to the identified pregnant women, gave them information about the study, obtained informed consent and then conducted the interview. Women were visited three times before they were considered unavailable to participate in the study.

### Data collection and quality control

There were 40 data collectors and four supervisors, all of whom had previous experience of data collection for other projects in the same district. The data collectors were trained for 2 days by the main study co-ordinator (TB) on administration of the instruments, the objectives of the study and ethical issues. Lectures, demonstrations and role plays were the methods used to train the data collectors. The main coordinator of the study also held weekly meetings with the data collectors and supervisors. The conduct of the study was closely monitored and supervised. Completed questionnaires were checked carefully on a daily basis for consistency, adherence to instructions and missing data, first by the supervisors, then by main coordinator and finally by the data entry clerks. Questionnaires deemed to have problems were returned back to the data collectors for investigation, and if necessary for correction or reassessment. Finally, data were computerized using a double data entry with EpiData version 3.1 (EpiData; CDC, 2000).

### Measurement

#### Outcomes

Utilisation of ANC services was assessed in relation to four dimensions of antenatal care: initiation of antenatal care (i.e., time of the first ANC visit), frequency of ANC attendance, adequacy of ANC attendance and adherence to ANC schedules.

Initiation of ANC and frequency of ANC attendance were assessed by an item in a self-report questionnaire asking about the gestational age at which the women attended for each of their ANC visits. Any ANC initiated before or at the 16th week of gestation was labeled as “timely initiation” and visits after this time were labeled “late” based on WHO recommendations. Adherence to ANC attendance schedules was estimated in relation to WHO guidance, which proposes that the first ANC attendance should take place before the 16th week of gestation; second ANC attendance between weeks 24 and 28; third ANC between weeks 30 and 32; and fourth ANC visit between weeks 36 and 40. Any visits out of these proposed intervals were considered to be non-scheduled. Women who initiated ANC visits between 24 and 28 weeks of gestation and continued the remaining visits as recommended were considered to be non-adherent for the first ANC schedules but adherent for the remaining ANC schedules. Finally, the numbers of scheduled and non-scheduled ANC visits were counted for each respondent. Respondents were asked about the number of emergency contacts for pregnancy complications with a range of specified types of traditional healers or biomedical health service providers available in the area [49].

The frequency of antenatal care visits was expressed as the ratio of the number of actual ANC visits to the total number of ANC visits recommended by WHO at the given gestational age. WHO proposes one, two, three and four ANC contacts for women at the 16th, 28th, 32nd and 40th week of gestation, respectively. Finally a ratio of 125 % or more of the recommended number of ANC visits was categorized as “increased use of ANC” and otherwise as “expected use of ANC”. Adequacy of ANC use was also categorized based on Kotlchuk’s index [50]. Women with 50 % or more ANC attendance and timely initiation (during or before the 16th week of gestation), as defined by WHO, were described as receiving “adequate ANC” while those with either late initiation or less than a 50 % expected attendance of ANC were defined as receiving “inadequate ANC”.

#### Exposure

Antenatal depressive symptoms were assessed with a locally validated Amharic version of the Patient Health Questionnaire (PHQ-9) [51]. A score of five or more was considered to be indicative of high antenatal depressive symptoms (the optimal score for increased probability of major depressive disorder in the criterion validation study). In Ethiopia, the PHQ-9 was found to have good internal consistency (Cronbach’s alpha = 0.81) and excellent intra-class correlation of 0.92 in a study of 926 outpatients in a major referral hospital in Addis Ababa [52]. Measures of depression specific to the perinatal period were considered; however, a validation study in rural Ethiopia concluded that Edinburgh Postnatal Depression Scale had low criterion validity and poor internal consistency [53]. Although previous Ethiopian studies had used the locally-validated Self-Reporting Questionnaire (SRQ-20) for measurement of common mental disorders [34], the PHQ-9 was preferred due to its focus on depressive symptoms [53].

#### Potential confounders

Intimate partner violence (IPV) was assessed using a five item scale, the Women’s Abuse Screening Test (WAST) [54, 55]. The scale was chosen for its brevity and the acceptability of the wording. Social support was measured using the Oslo Social Support Scale (OSSS-3) [56], a three item scale which asks about concern from others, ease of getting help and the number of supporting persons that participants can count on. Stressful life events were measured with the list of threatening experiences (LTE), a 12-item self-report questionnaire. The LTE has good test-retest reliability and internal consistency [57]. Both the OSSS-3 and LTE have been used in a community based study in the same setting [47].

Respondents were asked about the number of previous stillbirths, miscarriages, neonatal and infant mortality. The items were adapted from the Ethiopian Demographic Health Survey of 2011 [5]. Women were also asked whether they had chronic medical conditions, including HIV, tuberculosis, renal or cardiac diseases, hypertension, anemia or gastritis. Items asking the number of emergency health care provider visits to biomedical and traditional health care providers were used to assess women’s emergency health care use for pregnancy-related complications.

An item from the Ethiopian Demographic Health Survey was used to ask whether the woman wanted the pregnancy (labeled as “wanted”) or would have preferred it to happen at a future date (labeled as “mistimed”) or if she had never wanted to be pregnant at all (labeled as “unwanted”). Accessibility of health care was measured by using seven items asking respondents about the level of difficulty and distance to reach the nearest health facility and travel time to their respective nearest health facility, as well as affordability and availability of health care facilities [58]. Finally, self-reported pregnancy complications included a list of key danger signs during pregnancy, including bleeding, swollen hands/face, blurred vision, convulsions, high fever, loss of consciousness, severe abdominal pain, premature rupture of membranes, and discharge with unusual odor, pain during urination, severe headache and severe weakness. Closed ended questions were used to assess socio-demographic and socio-economic variables, including residence, marital status, estimated monthly income and educational level of participants.

### Data analysis

Data were analysed using the Statistical Packages for Social Sciences, version 20 (SPSS 20; IBM Corp 2012) and Stata version 13 (Stata Corp, 2013). A total of eight missing data [PHQ (1 missing), IPV (3 missing), accessibility to health care facility (3 missing) and LTE (1 missing)] were found. We included an average of observed scores of a respective variable to fill the missed item in the variables. Residence was categorized into urban/rural; marital status into married and single (never married, divorced and widowed) categories; Educational level into “non-literate”, “primary schooling (Grade 1–8)” and “Secondary Schooling” (Grade 9 and above) categories. Monthly income was categorized into tertiles as “high”, “medium”, and “low”. The profile of predictors and outcomes was described using simple descriptive summary values.

Poisson regression was used to examine the association between antenatal depressive symptoms and components of antenatal care use (number of non-scheduled and total number of ANC visits) after testing the assumption of equality of the variance and the mean. Negative binomial regression was used to examine the association between antenatal depressive symptoms and number of traditional and biomedical emergency health care provider visits. Socio-demographic and socioeconomic variables, interpersonal and life adversities, obstetric and medical conditions were included in all analyses as potential confounders. Gestational age was controlled as an offset variable in all poisson and negative binomial regression models. Binary logistic regression was used to test the relation between antenatal depressive symptoms and frequency, adequacy and initiation of antenatal health care use.

## Results

Amongst 1321 women identified as potentially eligible, 44 were excluded because they were in the first trimester of pregnancy; three refused participation and seven couldn’t be accessed despite three trials to find from their location.

### Characteristics of participants

Among study participants 98.6 % were married, 67.0 % were non-literate, and 92.1 % were rural residents. The mean age was 26.8 years (Table 1). With respect to ANC attendance, 60.5 % had initiated ANC (37.0 % by 16 weeks gestation). About one third of women fulfilled criteria for adequate antenatal care. Nearly 30.0 % of women had PHQ-9 score of five or more, indicating probable depression. Forty four percent of the participants reported an unintended pregnancy (36.2 % unwanted and 7.8 % mistimed) and 27.0 % had a history of adverse perinatal outcomes during their previous pregnancy.

**Table 1:** Characteristic of respondents (*N* = 1311)

Characteristics	Values	Number	Percent
Marital Status	^c^Single	18	1.4
	Married	1293	98.6
Pregnancy Intention	Unwanted	475	36.2
	Mistimed	102	7.8
	Wanted	734	56.0
Monthly Income category	High	429	32.7
	Medium	423	32.3
	Low	459	35.0
Residence	Rural	1208	92.1
	Urban	103	7.9
Mother’s Education	Grade 9–12 and above	53	4.0
	Grade 1–8	380	29.0
	Non-literate	878	67.0
Patient Health Questionnaire Status	PHQ ≥ 5	387	29.5
	PHQ < 5	924	70.5
Use of ANC during Past Pregnancy	Yes	793	60.5
	No	518	39.5
Number of Chronic Illnesses	One or more	440	33.6
	None	871	66.4
Past experience of adverse perinatal outcomes	1 or more	356	27.2
	None	955	72.8
Number of ANC Visits	Never initiated ANC	461	35.2
	Once or more	850	64.8
Self-reported pregnancy complications	None	655	50.1
	One or more	656	49.9
Adequacy of Antenatal Care	^a^Inadequate	861	65.7
	^b^Adequate	450	34.3
Time of Initiation of ANC	Timely (Up to 16th week of gestation)	455	37.7
	Late (After 16th week of gestation)	856	62.3

Mean age = 26.8 years

^a^Inadequate = less than 50 % of expected ANC attendance or late initiation

^b^Adequate = more than 50 % of expected ANC attendance and timely initiation

^c^Unmarried, divorced, widowed

### Antenatal depressive symptoms and adherence to ANC visits

Depressive symptoms during pregnancy were associated significantly with increased risk of having more non-scheduled ANC visits (aRR = 1.41, 95 % CI: 1.20, 1.65) and an increased number of total ANC visits (aRR = 1.23, 95 % CI: 1.12, 1.36) (Table 2).

**Table 2:** The association of antenatal depression with total and non-scheduled number of ANC visits

Characteristics		Total number of ANC Visits		Number of Non-scheduled ANC visits	
		CRR (95 % CI)	ARR (95 % CI)	CRR (95 % CI)	ARR (95 % CI)
Antenatal Depression:	PHQ-9 ≥ 5	1.12 (1.02, 1.23)^a^	1.23 (1.12, 1.36)^b^	1.21 (1.04, 1.40)^a^	1.41 (1.20, 1.65)^b^
	PHQ-9 < 5	1	1	1	1
Accessibility of Health facility		1.06 (1.03, 1.09)^a^	1.04 (1.02, 1.06)^b^	1.09 (1.04, 1.23)^b^	1.06 (1.02, 1.09)^b^
Marital Status:	Single	1.03 (0.72, 1.47)	0.99 (0.69, 1.42)	1.13 (0.60, 2.15)	1.04 (0.59, 1.82)
	Married	1	1	1	1
Residence:	Rural	0.65 (0.51, 0.83)^a^	0.85 (0.72, 1.01)	0.50 (0.40, 0.61)^b^	0.65 (0.51, 0.85)^b^
	Urban	1	1	1	1
Income:	High	1.15 (0.97, 1.37)	1.13 (1.00, 1.26)^a^	1.37 (1.11, 1.70)^a^	1.29 (1.07, 1.56)^a^
	Medium	1.06 (0.89, 1.26)	1.03 (0.92, 1.15)	1.13 (0.89, 1.43)	1.05 (0.88, 1.26)
	Low	1	1	1	1
Education:	Grade 9–12 and above	1.79 (1.28, 2.50)^b^	1.16 (0.92 (1.48)	2.06 (1.52, 2.81)^b^	0.99 (0.69, 1.43)
	Grade 1–8	1.27 (0.90, 1.49)	1.08 (0.96, 1.20)	1.13 (0.89, 1.43)	1.10 (0.92, 1.32)
	Non-literate	1	1	1	1
Intimate Partner Violence		1.01 (0.98, 1.03)	1.01 (0.99, 1.03)	1.00 (0.97, 1.03)	1.00 (0.97, 1.02)
Social Support		1.02 (0.99, 1.06)	1.02 (1.00, 1.04)	1.03 (0.99, 1.08)	1.03 (0.99, 1.07)
Number of Life threatening Events		1.01 (0.96, 1.06)	1.01 (0.98, 1.04)	1.05 (0.98, 1.17)	1.05 (1.00, 1.10)
Parity		0.93 (0.90, 0.96)^b^	0.94 (0.92, 0.97)^b^	0.89 (0.85, 0.94)^b^	0.91 (0.86, 0.95)^b^
Pregnancy Intention:	Unwanted	0.75 (0.65, 0.88)^b^	0.84 (0.75, 0.93)^b^	0.75 (0.61, 0.91)^a^	0.89 (0.75, 1.06)
	Mistimed	0.89 (0.68, 1.17)	0.91 (0.77, 1.09)	0.78 (0.56, 1.10)	0.86 (0.64, 1.15)
	Wanted	1	1	1	1
Adverse Perinatal outcomes: ≥1^c^		0.90 (0.77, 1.06)	0.94 (0.84, 1.04)	0.79 (0.64, 0.97)^a^	0.85 (0.71, 1.02)
ANC visit in past pregnancy: ≥1^c^		1.08 (0.94, 1.25)	1.19 (1.08, 1.31)^b^	1.10 (0.91, 1.33)	1.28 (1.09, 1.51)^a^
Chronic Medical conditions: ≥1^c^		1.08 (0.94, 1.25)	1.07 (0.97, 1.17)	1.14 (0.95, 1.37)	1.10 (0.95, 1.28)

^a^Statistically significant at < 0.05

^b^Statistically significant at < 0.001

^c^Reference group was “none”

Greater accessibility of the primary health care facility was associated with increased risk of having more non-scheduled ANC visits (aRR = 1.06 95 % CI: 1.02, 1.09) as well as with an increased number of total ANC visits (aRR = 1.04, 1.02, 1.06). Similarly, women with experience of ANC use in the past pregnancy had increased risk of more non-scheduled ANC visits (aRR = 1.28, 95 % CI: 1.09, 1.51) as well as total number of ANC visits (aRR = 1.19, 95 % CI: 1.08, 1.31) compared to those without experience of ANC use. Each increment in parity was associated with reduced risk of non-scheduled ANC visits (aRR = 0.91, 95 % CI: 0.86, 0.95) and a lower total number of ANC visits (aRR = 0.94, 95 % CI: 0.92, 0.97). Rural residence and low income were associated with reduced levels of non-scheduled ANC visits.

### Antenatal depressive symptoms and initiation, frequency and adequacy of ANC use

Antenatal depressive symptoms were associated with increased odds of ANC attendance beyond that recommended in ANC schedules (aOR = 1.86, 95 % CI: 1.29, 2.68). Increased access to the health facility (aOR = 1.16, 95 % CI: 1.07, 1.25) and experience of ANC in a previous pregnancy (aOR = 1.65, 95 % CI, 1.14, 2.41) were associated with increased ANC visits. Increasing parity (aOR = 0.84, 95 % CI: 0.75, 0.94) was associated with lower than expected ANC attendance. Increased parity (aOR = 0.90, 95 % CI: 0.83, 0.96) and unwanted pregnancy (aOR = 0.63, 95 % CI: 0.48, 0.83) were associated with lower levels of adequate ANC care (Table 3).

**Table 3:** The association of antenatal depression with initiation of ANC, frequency of ANC follow ups and adequacy of ANC

Characterstics		Initiation of ANC		ANC Visits higher than expected in a given Gestational Age		Adequacy of ANC Use	
		COR (95 % CI)	AOR (95 % CI)	COR (95 % CI)	AOR (95 % CI)	COR (95 % CI)	AOR (95 % CI)
PHQ 9 Score: PHQ-9 ≥ 5		1.10 (0.93, 1.24)	1.24 (0.95, 1.63)	1.45 (1.05, 2.05)^a^	1.86 (1.29, 2.68)^a^	1.09 (0.0.85, 1.39)	1.24 (0.94, 1.62)
		1	1	1	1	1	1
Access to Healthcare		1.10 (1.04, 1.15)^b^	1.07 (1.02, 1.13)^a^	1.18 (1.10, 1.27)^b^	1.16 (1.07, 1.25)^b^	1.10 (1.04, 1.15)^b^	1.07 (1.02, 1.13)^a^
Marital Status:	Married	1.20 (0.46, 3.12)	1.26 (0.47, 3.39)	1.31 (0.38, 4.58)	1.08 (0.29, 3.98)	0.96 (0.36, 2.56)	1.03 (0.37,2.86)
	Single	1	1	1	1	1	1
Residence: Rural		0.47 (0.31, 0.70)^b^	0.80 (0.48, 1.32)	0.39 (0.24, 0.62)^b^	0.62 (0.34, 1.14)	0.48 (0.32, 0.72)^b^	0.82 (0.49, 1.36)
Income:	High	1.14 (0.87, 1.51)	1.08 (0.80, 1.47)	1.19 (0.81, 1.75)	1.18 (0.77, 1.820)	1.16 (0.88, 1.54)	1.09 (0.80, 1.48)
	Medium	1.11 (0.84, 1.46)	1.05 (0.78, 1.40)	1.05 (0.71, 1.57)	0.97 (0.64, 1.47)	1.11 (0.84, 1.47)	1.04 (0.78, 1.40)
	Low	1	1	1	1	1	1
Education:	Non-literate	0.32 (0.18, 0.57)^b^	0.64 (0.32, 1.26)	0.39 (0.20, 0.76)^a^	1.06 (0.45, 2.50)	0.32 (0.18, 0.56)^b^	0.59 (0.29, 1.20)
	Elementary Schooling	0.46 (0.26, 0.82)^a^	0.61 (0.30, 1.23)	0.60 (0.30, 1.19)	1.10 (0.49, 2.47)	0.44 (0.25, 0.79)^a^	0.61 (0.31, 1.20)
	Secondary Schooling	1	1	1	1	1	1
Intimate Partner Violence		1.01 (0.97, 1.05)	1.02 (0.97, 1.06)	1.03 (0.99, 1.07)	1.02 (0.96, 1.08)	1.00 (0.97, 1.04)	1.01 (1.00, 1.06)
Social Support		1.04 (0.98, 1.10)	1.03 (0.97, 1.10)	1.00 (0.93, 1.09)	1.00 (0.92, 1.09)	1.04 (0.98, 1.04)	1.04 (0.97, 1.11)
Number of Life Threatening Events		1.04 (0.96, 1.13)	1.04 (0.95, 1.14)	1.04 (0.94, 1.16)	1.02 (.91, 1.16)	1.04 (0.96, 1.07)	1.04 (0.95, 1.14)
Parity		0.87 (0.82, 0.92)^b^	0.89 (0.83, 0.96)^a^	0.84 (0.77, 0.91)^b^	0.84 (0.75, 0.94)^a^	0.87 (0.82, 0.92)^b^	0.90 (0.83, 0.96)^a^
Pregnancy Intention:	Unwanted	0.54 (0.42, 0.69)^b^	0.65 (0.49, 0.86)^a^	0.62 (0.43, 0.88)^a^	0.78 (0.52, 1.15)	0.52 (0.40,0.67)^b^	0.63 (0.48, 0.83)^a^
	Mistimed	1.03 (0.67, 1.59)	1.11 (0.71, 1.71)	0.80 (0.43, 1.49)	0.84 (0.44, 1.60)	1.04 (0.68, 1.59)	1.11 (0.72, 1.72)
	Wanted	1	1	1	1	1	1
Previous Use of ANC: ≥1^c^		1.01 (0.80, 1.28)	1.14 (0.88, 1.47)	1.24 (0.89, 1.74)	1.65 (1.14, 2.41)^a^	1.04 (0.82, 1.31)	1.18 (0.91, 1.52)
Past Adverse obstetric history: ≥1^c^		0.98 (0.76, 1.26)	1.08 (0.82, 1.43)	0.72 (0.49, 1.06)	0.74 (0.49, 1.12)	0.96 (0.74, 1.24)	1.06 (0.80, 1.41)
Chronic Medical conditions: ≥1^c^		^a^1.30 (1.03, 1.64)	1.28 (1.00, 1.62)^a^	1.06 (0.77, 1.47)	1.03 (0.73, 1.45)	1.30 (1.03, 1.63)^a^	1.27 (1.00, 1.62)

^a^Statistically significant at < 0.05

^b^Statistically significant at < 0.001

^c^Reference was “none”

Antenatal depressive symptoms were not associated with timely initiation of ANC visits (aOR = 1.24, 95 % CI: 0.95, 1.63) (Table 3). Increased accessibility of the health facility (aOR = 1.07, 95 % CI: 1.02, 1.13) and having one or more chronic conditions (aOR = 1.28, 95 % CI: 1.00, 1.62) were associated significantly with increased timely initiation of ANC. Increased parity (aOR = 0.99, 95 % CI: 0.83, 0.96) and unwanted pregnancy (aOR = 0.65, 95 % CI: 0.49, 0.86) were associated significantly with delayed initiation of ANC.

### Antenatal depressive symptoms and emergency health care use

Antenatal depressive symptoms were associated with increased risk of a greater number of traditional emergency health care provider visits (aRR = 1.64, 95 % CI: 1.17, 2.31), biomedical health care provider visits (aRR = 1.32, 95 % CI: 1.04, 1.69) and increased number of total emergency health care visits (aRR = 1.44, 95 % CI: 1.16, 1.80) (Table 4).

**Table 4:** The association of antenatal depression and Number of traditional, modern and total number of emergency health care provider visits during pregnancy

Characteristics		Number of Traditional health care provider visits		Number of Modern health care provider visits		Number of either of both health care provider visits	
		cRR (95 % CI)	aRR (95 % CI)	cOR (95 % CI)	aRR (95 % CI)	cOR (95 % CI)	aRR (95 % CI)
PHQ 9 Score:	PHQ ≥ 5	2.15 (1.70, 2.71)^b^	1.64 (1.17, 2.31)^a^	1.29 (1.03, 1.62)^a^	1.32 (1.04, 1.69)^a^	1.63 (1.33, 1.99)^b^	1.44 (1.16, 1.80)^a^
	PHQ < 5	1	1	1	1	1	1
Access to Healthcare		0.94 (0.89, 0.98)^a^	1.07 (1.00, 1.14)	1.10 (1.06, 1.17)^b^	1.11 (1.06, 1.17)^b^	1.04 (1.00, 1.08)	1.08 (1.03, 1.13)^a^
Marital Status:	Single	0.19 (0.03, 1.37)	0.13 (0.01, 1.26)	1.17 (0.50, 2.74)	1.33 (0.56, 3.17)	0.75 (0.32, 1.72)	0.84 (0.35, 1.97)
	Married	1	1	1	1	1	1
Residence:	Rural	1.47 (0.86, 2.51)	3.87 (1.54, 9.74)^a^	060 (0.40, 0.92)^a^	0.79 (0.46, 1.34)	0.96 (0.71, 1.29)	1.17 (0.70, 1.95)
	Rural	1	1.00	1	1.00	1	1.00
Income:	High	4.44 (3.33, 5.93^a^	4.40 (2.89, 6.69)^b^	1.52 (1.15, 1.99)^a^	1.34 (1.00, 1.79)^a^	2.20 (1.72, 2.82)	1.93 (1.48, 2.51)^b^
	Medium	1.35 (0.95, 1.91)	1.97 (1.26, 3.08)^a^	1.00 (0.76, 1.32)	0.98 (0.74, 1.30)	1.11 (0.87, 1.43)	1.18 (0.91, 1.5)
	Poor	1	1	1	1	1	1
Education:	Secondary Schooling	0.98 (0.52, 1.84)	0.81 (0.25, 2.66)	1.50 (0.83 2.71)	0.96 (0.45, 2.06)	1.27 (0.73, 2.20)	0.92 (0.45, 1.88)
	Elementary Schooling	0.92 (0.72, 1.78)	1.07 (0.68, 1.7)	0.85 (0.66, 1.10)	0.86 (0.63, 1.16)	0.96 (0.77, 1.20)	0.96 (0.73, 1.28)
	Non-literate	1	1	1	1	1	1
Intimate Partner Violence		1.13 (1.10, 1.16)^b^	1.15 (1.09, 1.21)^b^	1.00 (0.96, 1.04)	0.99 (0.95, 1.03)	1.07 (1.04, 1.11)^b^	1.05 (1.02, 1.09)^a^
Social Support		0.96 (0.91, 1.01)	1.00 (0.92, 1.09)	1.04 (0.99, 1.10)	1.05 (0.99, 1.12)	1.01 (0.96, 1.06)	1.03 (0.97, 1.09)
Number of Life Threatening Events		1.30 (1.25, 1.350)^b^	1.27 (1.16, 1.39)^b^	1.10 (1.02, 1.17)^a^	1.07 (1.00, 1.16)	1.16 (1.09, 1.23)^b^	1.10 (1.03, 1.18)^a^
Parity		1.17 (1.11, 1.23)^b^	1.04 (0.94, 1.14)	1.04 (0.99, 1.10)	1.03 (0.95, 1.11)	1.07 (1.03, 1.13) ^b^	1.04 (0.98, 1.11)
Pregnancy Intention:	Unwanted	1.94 (1.53, 2.451)^b^	1.26 (0.86, 1.85)	0.95 (0.75, 1.21)	0.94 (0.72, 1.23)	1.17 (0.95, 1.44)	0.97 (0.76, 1.24)
	Mistimed	1.18 (0.76, 1.84)	0.72 (0.39, 1.34)	0.97 (0.64, 1.47)	0.98 (0.63, 1.51)	0.99 (0.68, 1.44)	0.89 (0.60, 1.32)
	Wanted	1	1	1	1	1	1
Past Adverse obstetric history: Yes^c^		0.71 (0.55, 0.92)^a^	0.53 (0.36, 0.80)^a^	1.10 (0.86, 1.40)	1.05 (0.81, 1.37)	0.97 (0.78, 1.21)	0.89 (0.70, 1.14)
Chronic Medical conditions: Yes^c^		3.51 (2.73, 4.51)^b^	2.86 (2.03, 4.03)^b^	1.58 (1.26, 1.98)^a^	1.46 (1.15, 1.84)^b^	2.14 (1.75, 2.63)^b^	1.84 (1.49, 2.28)^b^

^a^Statistically significant at < 0.05

^b^Statistically significant at < 0.001

^c^Reference was “none”

Having a chronic medical condition and higher income were associated with increased emergency health care visits of any type (traditional, biomedical and total number of health care visits). Increased number of traditional emergency health care provider visits was associated with rural residence (aRR = 3.87, 95 % CI: 1.54, 9.74), being in a high income category (aRR = 4.40, 95 % CI: 2.89, 6.69) and medium income categories (aRR = 1.07, 95 % CI: 1.26, 3.08), increased intimate partner violence (aRR = 1.15, 95 % CI: 1.09, 1.21), increased number of threatening life events (aRR = 1.27, 95 % CI: 1.16, 1.39) and having one or more chronic medical conditions (aRR = 2.86, 95 % CI: 0.03, 4.03). A history of adverse perinatal outcomes was associated with a lower risk of traditional health care visits (aRR = 0.53, 95 % CI: 0.36, 0.80) but not associated with emergency help-seeking from biomedical health care providers (aRR = 1.05, 95 % CI: 0.81, 1.37) (Table 4).

An increased number of biomedical health care provider visits was also associated with increased access to health care facility (aRR = 1.34 95 % CI: 1.00, 1.79), high income (aRR = 1.46, 95 % CI: 1.15, 1.84) and having one or more chronic medical conditions (aRR = 1.11, 95 % CI: 1.06, 1.17) as shown in Table 4.

## Discussion

In this community-based survey from rural Ethiopia, nearly one-third of antenatal women had high levels of depressive symptoms. Antenatal depressive symptoms were associated independently with increased number of non-scheduled ANC visits, and increased presentations for emergency health care. There was no association between antenatal depressive symptoms and initiation and adequacy of ANC.

### Prevalence of antenatal depressive symptoms

The prevalence of depressive symptom s among antenatal women varies across LMIC studies. Our finding of a relatively high prevalence of depressive symptoms (29.5 %) during pregnancy accorded with studies from South Africa [59], Ghana [31], Viet Nam [33] and south Brazil [60] where the prevalence of antenatal depression was 39, 26, 29.9 and 32.8 %, respectively. However, the prevalence of antenatal depressive symptoms in this study was higher than that seen in previous community studies from Ethiopia. In a study from south-western Ethiopia [32], which used the Edinburgh Postnatal Depression Scale (not validated for a rural Ethiopian population) the prevalence of antenatal depression was found to be 19.9 %. However, in a study in Butajira (a district on the southern border of Sodo) the prevalence of depressive and anxiety symptoms measured with the validated Self Reporting Questionnaire (SRQ-20) was found to be 12.0 % [34]. Normal symptoms of pregnancy which overlap with depressive symptoms in PHQ-9 may explain the relatively high prevalence of antenatal depressive symptoms in our study. Nonetheless, we have also noted the importance of somatic symptoms in the presentation of perinatal depression in this population [30]. Despite these variations, depressive symptoms in the perinatal period are important public health issues in LMICs, with the potential to affect not only health and functioning but also service use.

### Antenatal depression and ANC attendance

Our finding of increased non-scheduled ANC visits and total number of ANC visits in women with antenatal depressive symptoms were broadly consistent with the few reports from high income countries [25, 41, 61]. Increased non-scheduled ANC visits in our study is also in keeping with meta-analyses and systematic reviews of depression in general, where depression was associated with around a three times increase in non-adherence to medical recommendations [62, 63]. The increased non-scheduled ANC visits among women with depressive symptoms may be explained by somatic symptoms of depression motivating them to seek help. Our finding of an increased number of total ANC visits among women with depressive symptoms was mainly through non-scheduled ANC visits. The increased number of ANC visits among women with higher socio-economic status is in keeping with a study from Ghana [64].

### Antenatal depressive symptoms vs initiation and adequacy of ANC use

In the current study, antenatal depressive symptoms were not associated significantly with initiation of ANC visits in either univariate or multivariable models. This differs from the finding of delayed initiation of ANC in depressed women in the USA [42]. Timely initiation of ANC for some women might have preceded the onset of depressive symptoms since we recruited women in the second and third trimesters and consequently screened depressive symptoms after 16th week of gestation (a cut off time point of timely or delayed initiation of ANC) for most women. This might be the reason for the non-significant finding of the association between antenatal depressive symptoms and initiation of ANC in our study.

Antenatal depressive symptoms were associated with a higher overall number ANC visits than expected but non-significantly associated with adequacy of antenatal care visits after adjusting for potential confounders. The study replicated the non-significant association of antenatal depression and adequacy of antenatal health care visits in hospital based studies from the USA [41, 43].

### Antenatal depressive symptoms and emergency health care use

The finding of a significant association between antenatal depressive symptoms and number of emergency visits to traditional and biomedical health care provider visits is in keeping with hospital-based studies in Australia [65], Sweden [25] and the USA [41]. In those countries, antenatal depressive symptoms were associated with increased obstetrician visits and increased use of planned Caesarean section [25, 41].

Various explanations have been provided to understand increased emergency health care provider visits. First, reduced self-care [63], social support [66] and reduced cognition and motivation [67, 68] among people with depressive symptoms increase their vulnerability to other medical conditions and complications that consequently increase the chance of needing emergency intervention [63]. Second, women with depressive symptoms may delay timely help-seeking due to reduced social support [66], lower satisfaction with health care services [65], poorer motivation and memory of schedules [67, 68] and reduced self-care [63] which means that the condition is more likely to progress and lead to presentation as an emergency. Third, women with depression may worry more about perinatal symptoms [69–71] so that they seek emergency care. This has been reported previously in postnatal women in HICs, in which emergency healthcare utilization may be increased substantially, including visits to psychiatrists, paediatricians, general practitioners and traditional providers [65, 72].

Although selection bias was minimized in this population based study, recall bias is an important issue because of low literacy and poor record keeping in this predominantly rural population. Nevertheless, by conducting the study in the antenatal period, the risk of recall bias is reduced especially for measurement of the outcome variables. The risk of social desirability bias was minimized by training the data collectors so that they would clearly explain about the study and its objective to the participants before conducting interview, encouraging them to be frank in their responses. Though we used robust method to identify all cases during the study period, we cannot be certain whether all pregnant women in the district have been identified. Furthermore, we assessed women’s adherence to WHO’s recommendations on ANC attendance but not the adherence of health professionals to WHO’s recommendations to ANC.

## Conclusions

This study offers an insight to health planners about the potential effects of antenatal depressive symptoms on antenatal service utilization. Our finding of a strong association between antenatal depressive symptoms and use of ANC services, mostly through non-scheduled visits, is important, given the relatively high prevalence of depressive symptoms in this population. First, the limited service available for antenatal women could be overwhelmed. Secondly, the increase in non-scheduled visits could potentially be associated with undetected and untreated pregnancy complications as a result of inadequate antenatal care. Thirdly, depressive symptoms are associated with an increase in medically unexplained somatic complaints [63, 73], which may result in increased non-scheduled visits. Thus, establishing a system for improved detection, referral and treatments of antenatal depression may promote efficiency in the health system and may potentially reduce morbidity, mortality and cost.

## Abbreviations

ANC: Antenatal care
ANDS: Antenatal depressive symptoms
aOR: adjusted Odds Ratio
aRR: adjusted Risk Ratio
ARR: Adjusted Risk Ratio
CI: Confidence Interval
CRR: Crude Risk Ratio
GA: Gestational age
LMIC: Low and middle income countries
LMP: Last Menstrual period
